# Remarkable Effect Occasioned by a Sedative Poison

**Published:** 1812-09

**Authors:** Tho. Clark

**Affiliations:** No. 17, Denmark-street, Soho-square


*To the Editors of the Medical and Physical Journal.*

GENTLEMEN,

I CONSIDER it of importance to make known to the medical world, through the medium of the Medical and Physical

Physical Journal, a remarkable case, occasioned by a powerful *sedative poison*, which came under my observation, about two years since, when in France.

This case made the more lively impression on my mind, as upwards of thirteen years ago, while in the East Indies, I was eye-witness to a case of nearly a similar nature.

The person who was the subject of this last case publicly declared, that he firmly believed the disease with which he was then afflicted was caused by poison; however, very little credit was given to his assertions.

As it appears to me entirely foreign to the nature of your work to assign the probable reasons why such attempts had been made against the lives of innocent individuals, I shall say nothing more on that head.

The person in France against whose life an attempt was made by the *sedative poison* alluded to, was about forty years old, and of rather a delicate constitution. He received an invitation to breakfast from a married couple, whom he considered his most sincere friends; however, when he sat down to breakfast, the arrangement at table surprised him: a large cup of coffee was filled up for him alone; the husband poured a small quantity of milk into his own cup, but nothing whatever into his wife's cup, and she did not even sit down at table. From these and other collateral circumstances which occurred, he imagined that poison might be mixed with the coffee which was poured out for him alone. Still, however, he determined to taste it very sparingly, with a view to ascertain the true state of things; being of opinion that, tasting the coffee very sparingly, even if poison was mixed with it, could do him little or no injury.

The instant he tasted the coffee, however, his tongue and throat were slightly convulsed, the saliva was suddenly converted into a tough frothy matter, and his tongue clung to his mouth; and a sense of general debility, accompanied with slight tremors, instantaneously pervaded the whole system. At the same time he felt a peculiar sensation in the calves of his legs, which he could not well describe. His tongue was impressed with a rough, astringent, bitter, taste, accompanied with that peculiar diffusible sensation which is imparted to the organs of taste by certain kinds of essential oils. The taste was very much the same as that produced by chewing common laurel-leaves, only much stronger. He immediately declared that he was very ill. The husband recommended brandy—the wife said it was the sight of a piece of mutton on the table which made him sick!

The object of the person in question was now to get away as soon as possible, without giving reason to his *worthy hosts* to to suppose that he suspected them capable of such a horrid crime, being aware that there was not legal proof; besides, he thought it probable that another attempt of a similar nature might be made against his life, and in that event he would have endeavored to obtain legal proof.

As he was fully convinced that, if he swallowed any of the coffee it must have been a very small quantity indeed, as he tasted it very sparingly, and had some bread and butter in his mouth before, which he afterwards spat out, he did not think it necessary to take an emetic; being afraid of the debilitating effects of emetics, and being at the same time fully persuaded that the symptoms he then labored under would gradually cease.

During the whole of this day, the tremors and general debility, together with the peculiar sensation in the calves of his legs, and a scanty secretion of frothy saliva, continued to a greater or less degree. These symptoms were constantly alleviated by wine or exercise.

Next morning, as the tremors, &c. still continued, he took some castor-oil; however, he transacted his business as usual. Towards the evening of the same day, his tongue and throat were slightly swelled, and his voice somewhat hoarse, and the tremors and general debility were rather increased; but, after drinking a little of light wine, and taking supper, he felt himself tolerably well, and went to bed without any apprehensions of serious consequences. He did not sleep sound, and early the following morning he was suddenly seized with palpitations of the heart, and an extremely weak, frequent, and an irregular intermittent, pulse. A profuse cold sweat at the same time poured out from every part of his body, and he himself, as well as every person who saw him at that period, believed he was dying. He likewise had frequent slight spasmodic affections all over his body, but more especially of the alimentary canal and genital parts. The penis was much contracted and hard, but the scrotum much relaxed.

In the mean time he was covered very warmly, drunk a considerable quantity of wine, and his apartment was well warmed by means of a stove. In about two hours, the healthy temperature of his body returned. He still continued to perspire copiously, and it was only on the sixth day after the profuse sweating first began, that it ceased for an instant, though of different degrees of violence at different times. From time to time he had attacks of extreme debility, and was then threatened with fainting. During these paroxysms, the heart palpitated, and the pulse was frequent, irregular, and intermittent; but when these attacks of debility bility were absent, the pulse was tolerably regular, of good strength, and ranged from 86 to 96 in a minute, for about two days after the profuse sweating commenced; however, on the third and fourth days, the pulse ranged from 100 to 120, even when the paroxysms of debility did not exist.

For the first six days he drank from four to five bottles of weak light wine daily; and, whenever the attacks of debility came on, he insisted on having wine in considerable quantities, which constantly relieved him.

Although the profuse sweating began to cease at times on the ninth day, yet it was at least three weeks from its first commencement, until he passed one day without a sweating paroxysm; and these paroxysms were in the beginning constantly attended with palpitation of the heart, and a frequent, weak, irregular, pulse. The intervals between the paroxysms were at first short, but day after day they became longer, and, consequently, the paroxysms less frequent.

Notwithstanding, at the expiration of about three weeks from the commencement of the profuse sweating, the regular daily attacks of sweating ceased; still, however, for some months afterwards, he was occasionally subject to similar paroxysms. It is very singular that these paroxysms were never preceded by cold shiverings; neither did the person, during the whole of his illness, ever complain of general soreness and uneasiness, or any of the other symptoms that usually attend fevers. Indeed the only uneasy sensation that he ever complained of, was a sense of oppression in his chest, accompanied with a difficulty of breathing, and palpitation of the heart, when he labored under the fit of excessive weakness.

From the time the profuse perspirations broke out, he had a keen appetite, took large quantities of broth, and several eggs, daily, besides animal food occasionally. He likewise ate considerable quantities of fruit, particularly apples. His tongue was considerably swelled in the beginning of the disease, and of a dark color, and wine constantly moistened his mouth, and did not taste the same as it would have done if he had been in perfect health. His mental faculties were not at all impaired; on the contrary, they were preternaturally acute, and he scarcely, if at all, slept, until the profuse continued perspirations ceased at times. It is singular, that the sanguiferous system was extremely irritable, and readily obeyed the impulse of stimulants, when taken into the stomach, while at the same time the sensibility of the system at large was very much diminished, and (as the victim of this disease expressed himself,) he could have inflicted inflicted considerable wounds on himself without suffering much pain.

Notwithstanding the quantities of wine and other food which he constantly took during his illness, yet, at the expiration of about a week from the beginning of the profuse perspirations, he was so weak as to be scarcely able to stand; but, after this period, the profuse sweating having at times ceased, he gradually, though slowly, gained strength, and it was five weeks from the first attack before he could walk to any considerable distance. For several months afterwards he was subject to a difficulty of breathing, accompanied with a sense of oppression in his chest, and an extremely frequent weak pulse when he exerted himself considerably.

The perspiration was extremely volatile and offensive to the smell, and, in short, the whole body was in a putrescent condition.

During the whole of his illness his bowels were tolerably regular, the urine rather more abundant in general than natural, more especially during the first week of the disease. He took no medicines except wine, nourishing food, and fruit. However, when in a state of convalescence, he took from time to time decoction of bark, which invigorated the system, and relieved the difficulty of breathing, to which he was then subject, upon exerting himself considerably.

With regard to the case to which I was eye-witness in India, the person was about thirty years of age, and his health had been considerably impaired from repeated attacks of liver-complaint and dysentery.

One morning, while at breakfast, he was suddenly seized with a tendency to faint, and said that he believed himself poisoned, and requested a medical man, who was at table with him, to feel his pulse; who replied, that he did not know what was the matter with him, but that his pulse was extremely frequent and weak. He immediately drank some Madeira, and soon afterwards became better.

As the gentleman at breakfast with him drank of the tea which was on the table, it would seem that the poison had not been mixed with the tea; but, upon reflection, he thought it highly probable that the poison had been administered to him in some toddy, (a fermented liquor, obtained from the cocoa-nut tree,) which he drank about a quarter of an hour before. It seems to me extremely probable, that the stimulating effects of the fermented liquor had, as it were, counterbalanced, for a time, the sedative powers of the poison.

About

About two hours after he drank the Madeira, he was again threatened with fainting, which was likewise relieved by wine. He also had slight universal tremors, and spasmodic affections, from time to time, all over his body, but particularly in the alimentary canal, and organs of generation. The penis was much contracted and hard, and in the course of the first day he had several involuntary emissions of semen. The scrotum was much relaxed. The saliva was frothy, tenacious, and deficient in quantity. He likewise complained of a peculiar sensation in the calves of his legs.

In about thirty-six hours from the first attack, he became slightly deranged; however, he imagined no objects present that were not present, knew every body perfectly well who visited him, and reasoned on most subjects with tolerable propriety; but the most striking feature of his derangement was, that his attendants could make him believe almost any thing, however absurd, ridiculous, or inconsistent with sound judgment. His mind was uncommonly irritable and suspicious, and he did not sleep until the derangement ceased, which was at the expiration of a few days from the first attack.

In the mean time, he had frequent paroxysms of extreme debility, accompanied with palpitations of the heart, and a frequent weak intermittent pulse; and, when these attacks came on, he constantly supposed himself dying.

About two days from the beginning of the disease, he broke out into profuse sweat, and, in short, the symptoms throughout the whole of the disease were so similar to those already described in the former case, though generally more violent, that I think it unnecessary to enumerate them here; however, in the last case, while the person was deranged, the secretion of urine was uncommonly great, and seemed frequently mixed with purulent matter. Some of the medical men in attendance attributed this appearance of the urine to the probable absorption of purulent matter from the liver, which was no doubt then diseased.

The person who was the subject of the first case, remembers perfectly to have seen the husband bring out of his bedroom a tumbler *two-thirds* full of a colorless fluid, which he put into the coffee-pot, and which he said was cold water for clarifying the coffee; but, instead of carrying the tumbler in the usual manner, he covered its mouth with his hand: consequently, it seems to me highly probable, that this colorless liquor contained the poison, and that he had covered the tumbler with his hand in order to prevent its odor from being diffused.

 Hence,

Hence, as the taste of the poison, its immediate effect, and probably also its color, correspond entirely with those of laurel-water, is it not highly probable that the poison was really laurel-water?

Laurel-water is the only poison I know that operates instantaneously; and, when taken by the mouth, in the quantity of two or three spoonfuls, it produces almost instant death in the human species; and, when it is highly concentrated, death frequently takes place before the poison could have reached the stomach. Even when applied to external wounds of small animals it occasions death. It is also asserted that laurel-water produces similar effects when injected into the rectum.

I have not hitherto been able to acquire any satisfactory knowledge whatever with regard to the effects of laurel-water when taken in very small quantities; however, it seems highly probable, that even in very small doses it would produce very powerful changes in the human constitution; for, I am of opinion, that a poison so powerful as to occasion immediate death in the human species when taken in the quantity of one, two, or three, table-spoonfuls, must produce very important changes even when taken in very small doses.

It is said that the aquetta, or slow poison of the Italians, is so subtile in its nature, that, when administered in small quantities, its immediate effects are scarcely perceptible; yet, at the expiration of a few days, it occasions certain death. And it is also stated, that those who administer it can foretel almost precisely the period when the unhappy victim will die. Hence, there seems to me a striking analogy between the poison administered in the two foregoing cases, and the Italian slow poison.

Let it be supposed that a small quantity of a poison, similar to that used in the two preceding cases, is given to a person, in coffee, or otherwise, after having drank freely of any intoxicating liquor; it appears to me highly probable that the deleterious effect of the poison would be for a considerable length of time counteracted by the stimulating effect of the intoxicating liquor.

For instance, imagine one is invited to the house of a supposed friend to dine, and, after having drank freely of wine, or other inebriating liquors, a small quantity of the poison alluded to is administered to him, and that he goes to bed soon afterwards, it seems to me probable, that until the following morning the deleterious influence of the poison would not be at all evident. At this period he might perhaps complain of slight general tremors, and a sense of general debility, which symptoms most likely would be attributed to his having drank too freely the preceding evening; but, after these symptoms had continued for two or three days, if the person should then be seized with profuse cold sweating, attended with palpitations of the heart, and a weak, frequent, irregular, pulse, it seems likely that the case might be considered as an uncommon example of fever, accompanied with an affection of the chest; and, if the person should unfortunately die, dissection would show nothing satisfactory as to the cause of the disease.

Consequently, it seems probable, that the poison which had been administered in the two cases alluded to, was of the same kind with that which tyrants make use of in order to get rid of subjects whom they dread, and whom they cannot with safety to themselves either put to death or shut up in prison. However tyrannical a government may be, it is a dangerous experiment, even for the tyrants themselves, to cause men of well-known integrity to be put to death publicly, or to be imprisoned for life. Virtuous characters are regarded by them as highly-dangerous men, and enemies to their country. Hence, in such cases, subtile poison which can occasion death without giving rise to any suspicion whatever as to its cause, is the only sure resource by which these diabolical wretches can obtain the destruction of impartially just men.

I have the honor to be, Gentlemen,
With the utmost respect,
Your most obedient Servant,
THO. CLARK, M.D.

*No.* 17, *Denmark-street, Soho-square,*

*July* 12, 1812.

P. S. Since I did myself the honor of transmitting the above observations concerning two cases occasioned by powerful sedative poison, I have perused, with much pleasure and information, Mr. Brodie's Experiments concerning the Action of Vegetable Poisons, and find that there are many other substances besides cherry-bay laurel-water which produce death instantaneously.

Whatever the poison administered in the two cases alluded to might have been, it seems to have operated chiefly on the sanguiferous system; and the functions of the brain, instead of being diminished, became preternaturally acute.

It is true, in the case which happened in the East Indies, the person was slightly deranged for several days; but, although his ideas were erroneous on some subjects, yet, in other respects, he was uncommonly acute, and extremely suspicious.

I regret extremely that at present it is not in my power to try what effects might be produced on animals by administering small quantities of different kinds of poison, such as would not occasion immediate death.

It is well known that mercury, digitalis, or cicuta, are sometimes administered for a considerable length of time without producing considerable changes in the human constitution; and that these medicines, although their doses are not increased, yet sometimes suddenly produce violent effects.

Hence, I think it highly probable, that these poisons which are capable of occasioning sudden death, might, when administered in much smaller doses than would be necessary to occasion death, produce powerful changes in the animal economy, and, perhaps, in many instances, death itself, after a considerable elapse of time. T. C.

*July* 19, 1812.

